# Brain-Derived Neurotrophic Factor – A Major Player in Stimulation-Induced Homeostatic Metaplasticity of Human Motor Cortex?

**DOI:** 10.1371/journal.pone.0057957

**Published:** 2013-02-28

**Authors:** Claudia Mastroeni, Til Ole Bergmann, Vincenzo Rizzo, Christoph Ritter, Christine Klein, Ines Pohlmann, Norbert Brueggemann, Angelo Quartarone, Hartwig Roman Siebner

**Affiliations:** 1 Department of Neurology, Christian-Albrechts-University, Kiel, Germany; 2 Department of Neurosciences, Psychiatry and Anaesthesiological Sciences, University of Messina, Messina, Italy; 3 Donders Institute for Brain, Cognition and Behaviour, Radboud University Nijmegen, Njmegen, The Netherlands; 4 Section of Clinical and Molecular Neurogenetics at the Department of Neurology, University of Lübeck, Lübeck, Germany; 5 Department of Neurology, New York University School of Medicine, New York, New York, United States of America; 6 Department of Neurology, University of Hamburg, Hamburg, Germany; 7 Danish Research Centre for Magnetic Resonance, Copenhagen University Hospital Hvidovre, Hvidovre, Denmark; University of Toronto, Canada

## Abstract

Repetitive transcranial magnetic stimulation (rTMS) of the human motor hand area (M1_HAND_) can induce lasting changes in corticospinal excitability as indexed by a change in amplitude of the motor-evoked potential. The plasticity-inducing effects of rTMS in M1_HAND_ show substantial inter-individual variability which has been partially attributed to the val^66^met polymorphism in the *brain-derived neurotrophic factor* (*BDNF*) gene. Here we used theta burst stimulation (TBS) to examine whether the BDNF val^66^met genotype can be used to predict the expression of TBS-induced homeostatic metaplasticity in human M1_HAND_. TBS is a patterned rTMS protocol with intermittent TBS (iTBS) usually inducing a lasting increase and continuous TBS (cTBS) a lasting decrease in corticospinal excitability. In three separate sessions, healthy val^66^met (n = 12) and val^66^val (n = 17) carriers received neuronavigated cTBS followed by cTBS (n = 27), cTBS followed by iTBS (n = 29), and iTBS followed by iTBS (n = 28). Participants and examiner were blinded to the genotype at the time of examination. As expected, the first TBS intervention induced a decrease (cTBS) and increase (iTBS) in corticospinal excitability, respectively, at the same time priming the after effects caused by the second TBS intervention in a homeostatic fashion. Critically, val^66^met carriers and val^66^val carriers showed very similar response patterns to cTBS and iTBS regardless of the order of TBS interventions. Since none of the observed TBS effects was modulated by the BDNF val^66^met polymorphism, our results do not support the notion that the BDNF val^66^met genotype is a major player with regard to TBS-induced plasticity and metaplasticity in the human M1_HAND_.

## Introduction

The human motor cortex has a substantial potential to undergo plastic changes which may result in long-term potentiation (LTP)-like increases or long-term depression (LTP)-like decreases in corticospinal excitability [1], [2]. A wide range of transcranial stimulation protocols are capable of inducing lasting changes in corticospinal excitability in healthy human volunteers [3], including continuous or patterned repetitive transcranial magnetic stimulation (rTMS) [4], [5], [6], paired associative stimulation (PAS) [7], [8], continuous or oscillatory transcranial direct current stimulation [9], [10], transcranial alternating current stimulation [11], or transcranial random noise [12]. A frequently used patterned rTMS protocol is theta burst stimulation (TBS) which can induce bi-directional changes in corticospinal excitability in healthy human volunteers [5]. Intermittent TBS (iTBS) usually induces a lasting increase, whereas continuous TBS (cTBS) produces a lasting decrease in corticospinal excitability. When given sequentially, many of these protocols have been used to study homeostatic metaplasticity in the intact human motor cortex [3], [13], [14], [15]. Homeostatic metaplasticity refers to the phenomenon that level and even direction of stimulation-induced plasticity depend on the history of postsynaptic neuronal activity in the stimulated neuron population [3]. Previous LTP hampers further synaptic potentiation and facilitates depression whereas previous LTD has the opposite effect. This mechanism keeps synaptic plasticity in a functional range (see discussion for details). Stimulation-induced LTP-like, LTD-like, or homeostatic plasticity can be readily assessed with single-pulse transcranial magnetic stimulation (TMS) by measuring changes in mean amplitude of the motor evoked potential (MEP) in contralateral hand muscles [14], [15], [16], [17], [18].

It has been suggested that plasticity-inducing stimulation protocols can be used therapeutically to improve motor function in patients with motor stroke or movement disorders [19], [20], [21], [22], [23]. However, one major limitation of present stimulation protocols is that the after effects on motor cortex excitability are highly variable across individuals. This may in part be due to inter-individual differences in the ability of cortical neurons to express synaptic plasticity. Indeed, neurobiological factors such as the phase of the menstrual cycle in women [24], [25] or circadian changes in circulating cortisol levels [26] appear to influence the individual responsiveness of the human motor cortex to transcranial stimulation.

Genetic factors contribute to the inter-individual variability in stimulation-induced plasticity [27]. In a recent twin study, the heritability of PAS-induced motor cortex plasticity was estimated to be 0.68 based on the intra-pair difference of LTP-like PAS effects in monozygotic and dizygotic twins [28]. Several studies in human and mice have provided converging evidence that LTP formation, memory, and motor learning are influenced by the val^66^met single nucleotide polymorphism in the brain derived neurotrophic factor (BDNF) gene [29], [30], [31]. For instance, individuals carrying the val^66^met polymorphism in the BDNF gene show less increase in MEP amplitude after motor training than val^66^val carriers [29], [30]. BDNF has been implicated in the control of NMDA receptor-dependent synaptic plasticity and its homeostatic regulation [32]. Animal studies have shown that mature BDNF (mBDNF) plays an important role in all stages of long-term potentiation (LTP), whereas its precursor peptide (pro-BDNF) has been associated with long-term depression (LTD) [33], [34].

Cheeran and colleagues (2008) were the first to study the impact of val^66^met polymorphism in the BDNF gene on stimulation-induced plasticity in the human motor hand area (M1_HAND_). Compared to nine val^66^val carriers, nine age-and sex-matched val^66^met carriers showed a marked attenuation of LTP-like plasticity in response to iTBS and PAS as well as reduced LTD-like plasticity in response to cTBS [29]. Further, cathodal transcranial direct current stimulation (tDCS) did not trigger a homeostatic response to subsequent 1Hz rTMS in eight val^66^met carriers, whereas eight age- and sex-matched val^66^val carriers showed the expected homeostatic reversal of corticospinal excitability towards facilitation [29]. Prompted by these results, several other groups evaluated the effect of the BDNF val^66^met genotype on the direction and magnitude of stimulation-induced corticospinal excitability using different interventional protocols [28], [29], [30], [35], [36], [37], [38], [39]. Since these studies yielded inconsistent results, this study was designed to reexamine the influence of the val^66^met BDNF genotype on LTP-like and LTD-like plasticity, as well as homeostatic metaplasticity. To exclude any examiner bias, participants and examiner were completely blind to the genotype of the participants. Like in the seminal study by Cheeran et al. [29], we chose iTBS and cTBS as interventional protocols to assess the effect of the val^66^met BDNF genotype on LTP-like and LTD-like plasticity, respectively. In contrast to Cheeran et al. [29], we also used iTBS and cTBS to probe the effect of the val^66^met BDNF genotype on the individual expression of homeostatic metaplasticity. To this end, all subjects underwent three different interventions: cTBS followed by cTBS (c-cTBS), cTBS followed by iTBS (c-iTBS), and iTBS followed by iTBS (i-iTBS). While our interventions reliably induced LTP-like and LTD-like plasticity as well as homeostatic metaplasticity, we found no evidence for a significant influence of BDNF polymorphism on TBS induced plasticity.

## Methods

### Subjects

Twenty-nine healthy right-handed male volunteers (mean age 26.0±3.2 SD) were recruited from the student population of the University of Kiel. Participants had no history of neurological disease and did not take CNS-active medication at the time of the study. All participants had previously participated in TMS studies but where naïve to the specific purpose of our study. Subjects participated after they had given written informed consent. Experimental procedures conformed to the Declaration of Helsinki and were approved by the local Ethics Committee of the University of Kiel. The sample was confined to male subjects since hormonal fluctuations associated with the female cycle are known to strongly modulate cortical plasticity and intracortical inhibition [24] and we decided to exclude this additional source of variance from our data.

### Experimental Procedures

**Figure 1:**
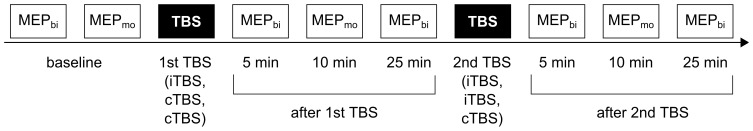
Time line of an experimental session. The experiment consisted of three of these sessions in each of which two different TBS protocols were subsequently applied: iTBS followed by iTBS (i-iTBS), cTBS followed by iTBS (c-iTBS), and cTBS followed by cTBS (c-cTBS).Motor evoked potentials (MEPs) were recorded at the contralateral FDI muscle both with biphasic pulses (MEP_bi_) at baseline as well as 5 and 25 minutes after the end of each TBS intervention and with monophasic pulses (MEP_mo_) at baseline, and 10 min after the end of each TBS intervention. Session were randomized in order across subjects and were conducted at least five days apart.

The TMS experiments consisted of three separate sessions performed at least five days apart to minimize carry-over effects (Fig. 1). All experiments were performed during day time (10∶00 am–7∶00 pm) hours. In each session, subjects received two TBS interventions to the left M1_HAND_ which were separated by an interval of ∼30 minutes. Apart from two subjects, all participants received three different combinations of TBS (Fig. 1): (i) cTBS followed by cTBS (c-cTBS), (ii) iTBS followed by iTBS (i-iTBS), and (iii) cTBS followed by iTBS (c-iTBS). The order of TBS-TBS interventions was pseudorandomized across subjects. One subject only participated in the c-iTBS session and another subject only in the c-iTBS and i-iTBS session. At the beginning of the first session, a blood sample was taken for BDNF genotyping.

### Transcranial Magnetic Stimulation

TMS was performed with a standard figure-of-eight coil with outer diameter of 100 mm “MC-B70” and a MagPro-X100 stimulator (Magventure, Skovlunde, Denmark). The coil was placed over the left M1_HAND_ tangentially to the skull and with the handle pointing backwards and laterally at an angle of ∼45° to the sagittal plane. The first phase of the monophasic stimulus and the second (reversal) phase of the biphasic stimulus had an anterior-medial to posterior-lateral (a-p) direction in the coil. Hence, each TMS pulse induced a maximal current flow in the brain tissue with a posterior-lateral to anterior-medial (p-a) direction.

Before starting with the experiment we determined in each session the optimal coil position for evoking maximal MEPs in the right first dorsal interosseus (FDI) muscle (referred to as “motor hot spot”). We then defined the resting motor threshold (RMT). RMT was defined as the minimum intensity evoking a peak-to-peak MEP of 50 µV in 5 of 10 consecutive trials in the relaxed FDI muscle. The active motor threshold (AMT) was defined as the minimum intensity that elicited a reproducible MEP of at least 200 µV in the tonically contracted FDI muscle in 5 out of 10 consecutive trials using a biphasic pulse. Participants were asked to produce a force level of 10% of maximal voluntary contraction.

Corticospinal excitability is commonly probed using monophasic stimuli, whereas TBS usually consists of biphasic stimuli. In this study, we assessed corticospinal excitability with single-pulse TMS using both, a monophasic (MEP_mo_) as well as biphasic (MEP_bi_) pulse configuration. To obtain a reliable estimate of mean MEP amplitude, corticospinal excitability was measured in blocks of 30 MEPs immediately before the first TBS conditioning (referred to as baseline) and after the end of each TBS conditioning: twice with biphasic pulses (5 and 25 min post-TBS) and once with monophasic pulses (10 min post-TBS) (Fig. 1). Stimulation intensity for MEP measurements was adjusted separately for monophasic and biphasic pulse mode to evoke an MEP of approximately 0.5 mV peak-to-peak amplitude at baseline with each pulse form. The interstimulus interval between two consecutive TMS pulses was 5 s.

TBS was performed according to the original protocol described by Huang et al. [5]. Three biphasic TMS pulses were given as short 50 Hz bursts every 200 ms. For cTBS, TBS was continuously applied as a 40 s train. For iTBS, a single TBS train lasted 2 s and was repeated every 10 s for a total of 190 s. The total number of TMS stimuli was identical for cTBS and iTBS (600 stimuli). Stimulation intensity was individually adjusted to 80% of AMT.

Coil position and orientation was kept in a constant position with the help of frameless stereotaxy (Localite TMS Navigator, St. Augustin, Germany) after coregistration of individual T1-weighted whole brain magnetic resonance images. The T1-weighted images were acquired several days before the first experimental TMS session (170 sagittal slices, 1×1×1 mm isotropic voxel size, field- of-view 224×224 mm). MRI was performed on a 3-Tesla MRI system using an 8-channel head coil (Philips Achieva, Philips Medical Systems, Best, The Netherlands) and a standard magnetization prepared rapid acquisition gradient echo (MPRAGE) sequence (TR: 7.7 ms, TE: 3.6 ms, flip angle: 8°).

### Recording of Motor-evoked Potentials

Surface EMG activity was recorded from the FDI muscle with Ag/AgCl electrodes which were attached to the skin using a bipolar belly-tendon montage. EMG signal was amplified (1000×) and band-pass filtered (1 Hz to 1 KHz) (D360, Digitimer, Welwyn Garden City,Herts, UK), digitized at a rate of 5 kHz on a trial-by-trial basis (CED Power1401 interface and Signal2 software, Cambridge Electronic Design, Cambridge, UK), and stored on a personal computer for off-line analysis. Auditory feedback of background EMG activity was continuously provided to help participants to completely relax or to maintain a constant level of contraction during the calculation of AMT. Peak-to-peak MEP amplitudes of the right FDI muscle were measured off-line on a trial-by-trial basis and then averaged for each block of measurement (NuCursor software, Sobell Research Department of Motor Neuroscience and Movement Disorders, Institute of Neurology, University College of London, UK).

### Genotyping of the val66met BDNF Genotype

After obtaining informed consent, EDTA blood samples were collected and genomic DNA was extracted. Genotyping of a common polymorphism in the BDNF gene (p.V66M, c.196G>A) was performed by a DNA melting curve analysis with variant-specific probes on a LightCycler device (Roche Diagnostics, Mannheim, Germany) following polymerase chain reaction. Primer sequences and PCR conditions are available on request. The authors were only unblinded towards the subjects’ genotypes after they had completed data acquisition and data analysis at the within-subject level to avoid any examiner bias.

### Statistical Analyses

Data were analyzed using SPSS V. 13.0. One-sided one-sample t-tests were used to test for the expected increases in MEP amplitude after iTBS and decreases in MEP amplitude after cTBS. Statistical comparisons between TBS protocols, time points of measurement, and BDNF polymorphism were based on mixed ANOVAs. Greenhouse-Geisser correction for non-sphericity was applied conditional on a significant Mauchly’s test. Post-hoc two-sided paired-sample t-tests were performed if applicable. P-values <0.05 were considered significant. Data are presented as mean ± SD if not specified otherwise.

To rule out any potentially confounding differences at pre-TBS baseline, we first calculated four separate mixed two-way ANOVAs (n = 27) with the within-subject factor type of sequential *TBS protocol* (c-cTBS, i-iTBS, c-iTBS) and the between-subject factor *polymorphism* (val^66^val, val^66^met) using RMT, AMT, baseline MEP_bi_ amplitude, and baseline MEP_mo_ amplitude as the respective dependent variable.

The first set of main analyses investigated the differential time courses of corticospinal excitability changes for the three sessions and their dependency on the BDNF polymorphism, with a particular focus on the after-effects of the first TBS intervention. Analyses were performed separately for mean MEP amplitudes elicited with monophasic (MEP_mo_) or biphasic pulses (MEP_bi_). Mean MEP amplitudes after the first TBS intervention were baseline-adjusted and expressed as percentage of the mean MEP amplitude measures at pre-TBS baseline. One-sided one-sample t-tests were used to test whether iTBS induced significant increases and cTBS significant decreases in corticospinal excitability. For MEP_bi_ we performed a three-way mixed ANOVA (n = 27) with the within-subject factors *protocol* (c-cTBS, i-iTBS, c-iTBS) and *time* (5 min after 1^st^ TBS, 25 min after 1^st^ TBS, 5 min after 2^nd^ TBS, 25 min after 2^nd^ TBS) and the between-subject factor *polymorphism* (val^66^val, val^66^met). For MEP_mo_ we performed a three-way mixed ANOVA (n = 27) with the within-subject factors *protocol* (c-cTBS, i-iTBS, c-iTBS) and *time* (10 min after 1^st^ TBS, 10 min after 2^nd^ TBS) and the between-subject factor *polymorphism* (val^66^val, val^66^met).

The second set of main analyses assessed whether the after-effects of TBS were homeostatically modulated by a preceding TBS and whether any homeostatic effects depended on the BDNF polymorphism. Please note that MEP amplitudes were now adjusted relative to the MEP measurement directly preceding the respective TBS intervention (calculated as percent of this new baseline) to correct for any MEP facilitation or suppression already induced by the first TBS intervention. One-sided one-sample t-tests evaluated whether the differentially preconditioned iTBS/cTBS protocols induced actual increases/decreases from their respective baselines. Then, we compared the after-effects of iTBS conditioned by iTBS (iTBS_iTBS_; 2nd TBS in i-iTBS session), by cTBS (iTBS_cTBS_; 2nd TBS in c-iTBS session), and by no TBS (iTBS_noTBS_; 1st TBS in i-iTBS session). In parallel we compared the after-effects of cTBS conditioned by cTBS (cTBS_cTBS_; 2nd TBS in c-cTBS session) and by no TBS (cTBS_noTBS_; 1st TBS in c-cTBS session). These analyses were performed separately for iTBS and cTBS and for the dependent variables MEP_mo_ and MEP_bi_. For MEP_bi_ we performed two separate three-way mixed ANOVAs (n = 28) with the within-subject factors *preconditioning* (iTBS_iTBS_, iTBS_cTBS_, iTBS_noTBS_) or (cTBS_cTBS_, cTBS_noTBS_) and *time* (5 min after iTBS, 25 min after iTBS) and the between-subject factor *polymorphism* (val^66^val, val^66^met). For MEP_mo_ we performed two separate two-way mixed ANOVAs (n = 27) with the within-subject factors *preconditioning* (iTBS_iTBS_, iTBS_cTBS_, iTBS_noTBS_) or (cTBS_cTBS_, cTBS_noTBS_) and the between-subject factor *polymorphism* (val^66^val, val^66^met) for the MEP_mo_ measurement 10 min after iTBS.

## Results

Regarding the BDNF genotype, 12 participants were val^66^met allele carriers, while 17 participants were val^66^val allele carriers, and no subject was a met^66^met carrier. No subject experienced any noticeable adverse affect during the course of the study other than mild local discomfort at the site of TBS. At pre-TBS baseline, neither RMT (33.5±5.4% MSO), AMT (25.0±5.5% MSO) nor mean MEP_bi_ amplitude (0.61±0.10 mV) and mean MEP_mo_ amplitude (0.61±0.15 mV) differed between sessions or BDNF genotypes (all P>0.2).

### General Effects of iTBS and cTBS on Corticospinal Excitability

**Figure 2:**
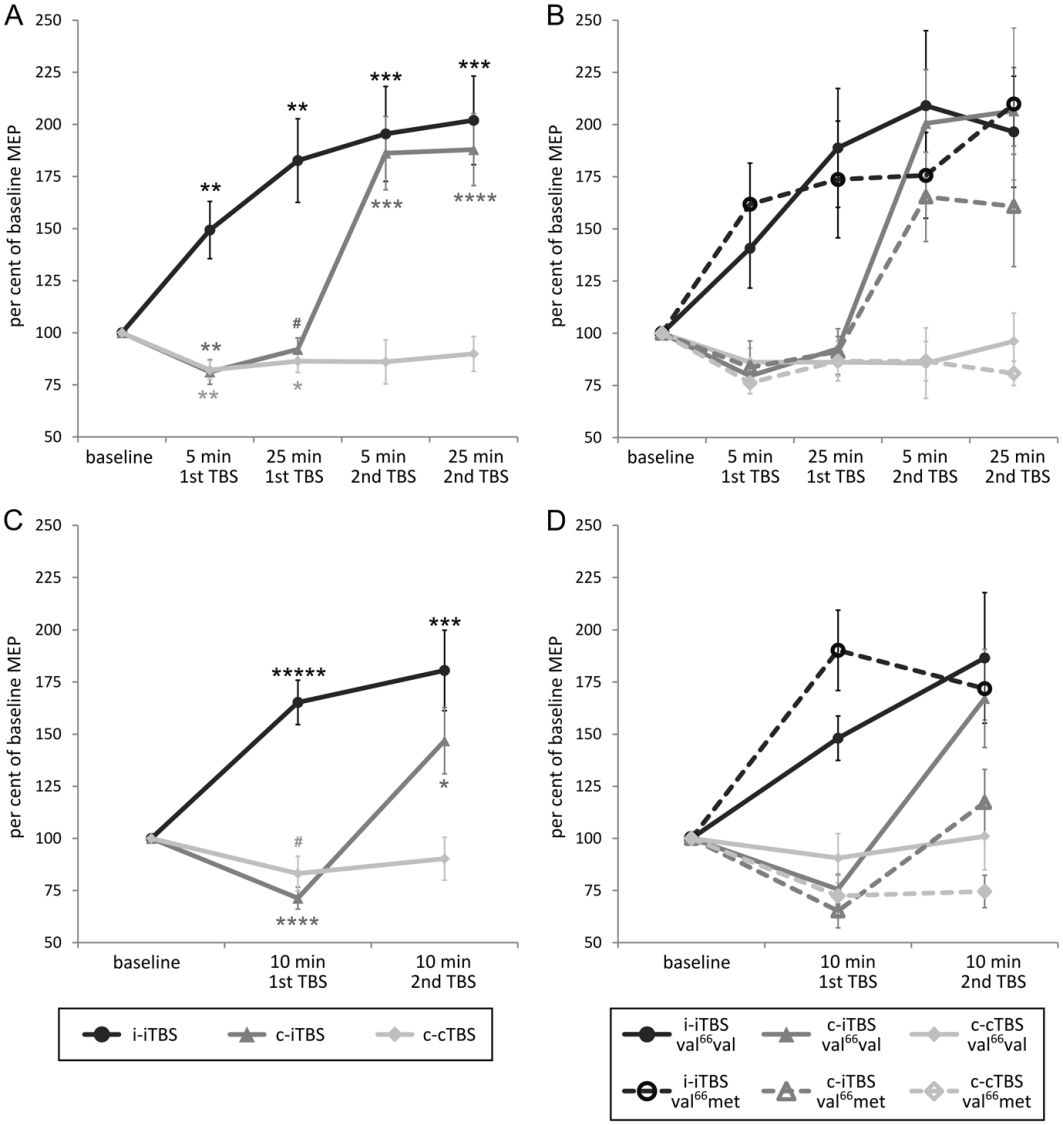
Effects of the three TBS-TBS protocols and BDNF polymorphism on corticospinal excitability over time: (A) MEP_bi_ amplitude independent of polymorphism, (B) MEP_bi_ amplitude divided by polymorphism, (C) MEP_mo_ amplitude independent of polymorphism, (D) MEP_mo_ amplitude divided by polymorphism. Asterisks indicate significant changes from baseline in the expected direction as revealed by one-sided one-sample t-tests (^#^P<0.05, *P<0.01, **P<0.001, ***P<0.0001, ****P<0.00001, *****P<0.000001; please note that p-values indicated by one or more asterisks are also significant when applying two-sided t-tests).

Figure 2 displays MEP amplitude throughout all TBS-TBS sessions as percent of the initial baseline measurements (note that all statistics in this section also rely on these percent values). One-sampled t-tests confirmed that iTBS consistently induced an increase and cTBS consistently induced a decrease in both MEP_bi_ and MEP_mo_ amplitude relative to pre-TBS baseline. In the i-iTBS session, mean MEP_bi_ amplitude was facilitated after the 1^st^ iTBS (5 min: T_27_ = 3.42, P = 0.001; 25 min: T_27_ = 3.97, P<0.001) as well as after the 2^nd^ iTBS (5 min: T_27_ = 4.33, P<0.0001; 25 min: T_27_ = 4.76, P<0.0001). In the c-iTBS session, mean MEP_bi_ amplitude was reduced after the 1^st^ cTBS (5 min: T_28_ = 3.52, P<0.001; 25 min: T_28_ = 1.81, P<0.04) and facilitated after the 2^nd^ iTBS (5 min: T_28_ = 4.68, P<0.0001; 25 min: T_28_ = 5.33, P<0.00001) relative to pre-TBS baseline. In the c-cTBS session, mean MEP_bi_ amplitude decreased after the 1^st^ cTBS (5 min: T_26_ = 3.92, P<0.001; 25 min: T_26_ = 2.51, P<0.01) but did not differ from baseline after the 2^nd^ cTBS (5 min: P = 0.1; 25 min: P>0.1). The same pattern was evident when the mean MEP_mo_ amplitude was used as index of corticospinal excitability. In the i-iTBS session, mean MEP_mo_ amplitude was facilitated after both the 1^st^ iTBS (10 min: T_27_ = 6.10, P<0.000001) and the 2^nd^ iTBS (10 min: T_27_ = 4.35, P<0.0001). In the c-iTBS session, mean MEP_mo_ amplitude was attenuated after the 1^st^ cTBS (10 min: T_28_ = 5.77, P<0.00001) and facilitated after the 2^nd^ iTBS (10 min: T_28_ = 3.03, P<0.01). In the c-cTBS session, mean MEP_mo_ amplitude was reduced after the 1^st^ cTBS (10 min: T_26_ = −2.03, P<0.05) but showed no difference after the 2^nd^ cTBS (10 min: P>0.1).

ANOVAs for both, MEP_bi_ and MEP_mo_ amplitudes, revealed a main effect of *protocol* (MEP_bi_: F_(1.4,35.4)_ = 25.91, P<0.00001; MEP_mo_: F_(2,50)_ = 24.3, P<0.0000001) and *time* (MEP_bi_: F_(1.8,46.1)_ = 10.51, P<0.001; MEP_mo_: F_(1,25)_ = 9.36, P<0.01) as well as an interaction of *protocol*×*time* (MEP_bi_: F_(4.2,106.9)_ = 7.82, P<0.00001; MEP_mo_: F_(2,50)_ = 7.54, P<0.01) but no impact of *polymorphism* (all P>0.2; despite a P = 0.081 trend for the polymorphism*time interaction in MEP_mo_ only).

### Priming the Plasticity-inducing Effects of iTBS

**Figure 3:**
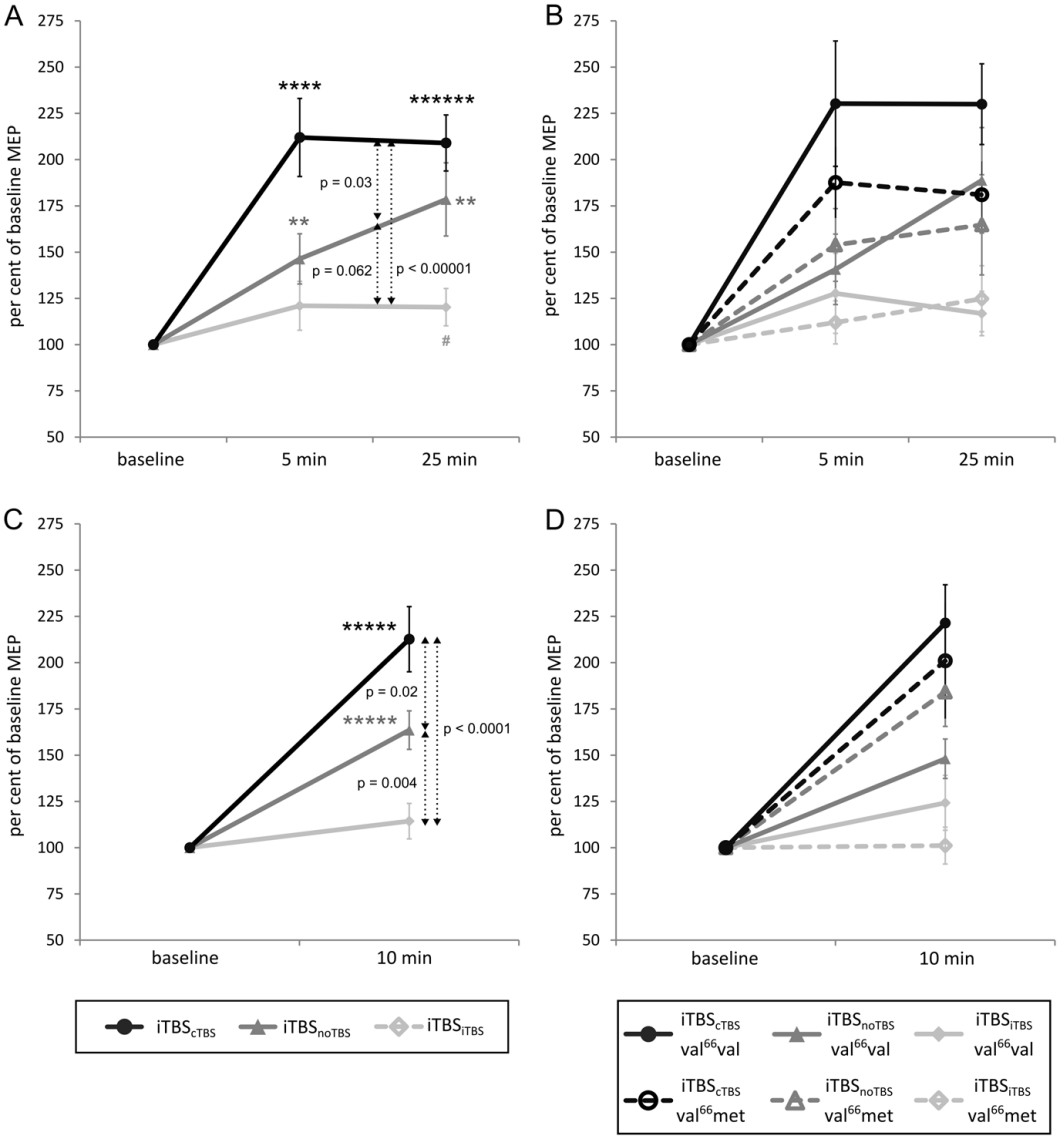
Effect of preconditioning on the after-effects of iTBS: (A) MEP_bi_ amplitude independent of polymorphism, (B) MEP_bi_ amplitude divided by polymorphism, (C) MEP_mo_ amplitude independent of polymorphism, (D) MEP_mo_ amplitude divided by polymorphism. Asterisks indicate significant changes from baseline in the expected direction as revealed by one-sided one-sample t-tests (^#^P<0.05, *P<0.01, **P<0.001, ***P<0.0001, ****P<0.00001, *****P<0.000001, ******P<0.0000001; please note that p-values indicated by one or more asterisks are also significant when applying two-sided t-tests). Actual p-values are given for post-hoc two-sided paired t-tests comparing different conditions.

Figure 3 displays the effects of iTBS as percent of the respective immediate pre-iTBS baseline (note that all statistics in this section also rely on these percent values). The after-effects of iTBS strongly depended on preconditioning. One-sample t-tests confirmed the principal facilitation of corticospinal excitability after iTBS. When iTBS was preconditioned with cTBS (iTBS_cTBS_), both MEP_bi_ (5 min: T_28_ = 5.13, P<0.00001; 25 min: T_28_ = 7.44, P<0.0000001) and MEP_mo_ (10 min: T_28_ = 6.30, P<0.000001) were markedly facilitated. The same held true for unconditioned iTBS (iTBS_noTBS_): MEP_bi_ (5 min: T_27_ = 3.42, P = 0.001; 25 min: T_27_ = 3.97, P<0.001) and MEP_mo_ (10 min: T_27_ = 6.10, P<0.000001). However, when iTBS was preconditioned with iTBS (iTBS_iTBS_), facilitation was less strong for both MEP_bi_ (5 min: T_27_ = 1.59, P = 0.062; 25 min: T_27_ = 2.00, P = 0.027) and MEP_mo_ (10 min: T_27_ = 1.49, P = 0.073).

Accordingly, ANOVAs revealed a main effect of *preconditioning* for both MEP_bi_ (F_(2,52)_ = 9.85, P<0.001) and MEP_mo_ amplitude (F_(2,52)_ = 13.94, P<0.0001) but no impact of *time* (for MEP_bi_) or *polymorphism* (all P>0.2). Post-hoc comparisons showed that facilitation was boosted for iTBS_cTBS_ relative to both iTBS_noTBS_ (MEP_bi_: T_27_ = 2.28, P = 0.03; MEP_mo_: T_27_ = 2.48, P = 0.02) and iTBS_iTBS_ (MEP_bi_: T_27_ = 6.17, P<0.00001; MEP_mo_: T_27_ = 4.86, P<0.0001), whereas facilitation after iTBS_iTBS_ was tendentially reduced relative to iTBS_noTBS_ for MEP_bi_ (T_27_ = 1.94, P = 0.064) and clearly suppressed for MEP_mo_ (T_27_ = 3.14, P<0.01).

### Priming the Plasticity-inducing Effects of cTBS

**Figure 4:**
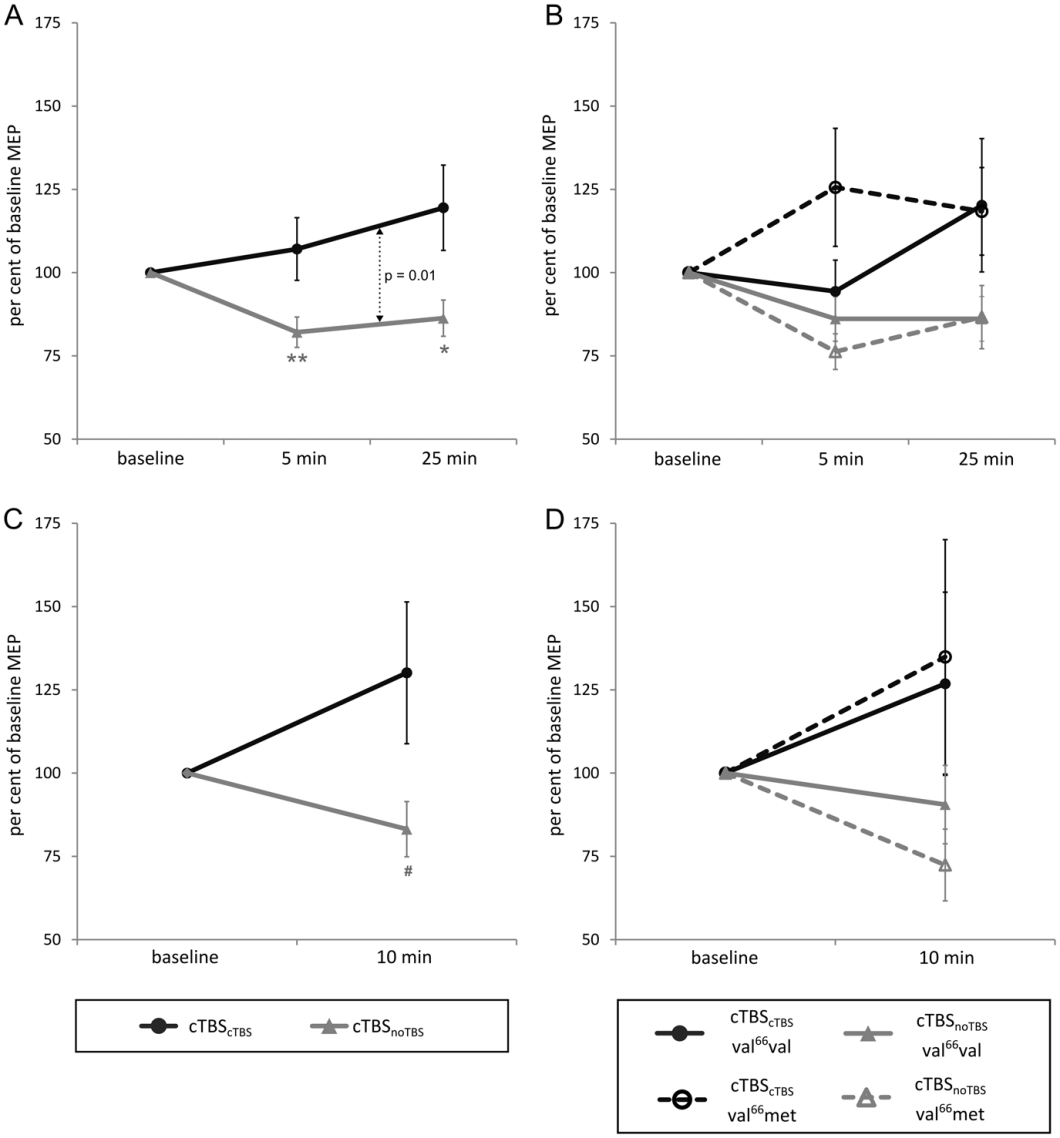
Effect of preconditioning on the after-effects of cTBS: (A) MEP_bi_ amplitude independent of polymorphism, (B) MEP_bi_ amplitude divided by polymorphism, (C) MEP_mo_ amplitude independent of polymorphism, (D) MEP_mo_ amplitude divided by polymorphism. Asterisks indicate significant changes from baseline in the expected direction as revealed by one-sided one-sample t-tests (^#^P<0.05, *P<0.01, **P<0.001; please note that p-values indicated by one or more asterisks are also significant when applying two-sided t-tests). Actual p-values are given for post-hoc two-sided paired t-tests comparing different conditions.

Figure 4 displays the effects of cTBS as percent of the respective immediate pre-cTBS baseline (note that all statistics in this section also rely on these percent values). Also the after-effects of cTBS depended on preconditioning. One-sample t-tests confirmed the principal inhibition of corticospinal excitability after cTBS when it was unconditioned (cTBS_noTBS_) for both MEP_bi_ (5 min: T_26_ = 3.92, P = 0.001; 25 min: T_26_ = 2.51, P<0.01) and MEP_mo_ (10 min: T_26_ = 2.03, P<0.05). However, when conditioned with cTBS (cTBS_cTBS_) neither MEP_bi_ (5 min: P>0.7; 25 min: P>0.9) nor MEP_mo_ (10 min: P>0.9) showed any reduction in mean MEP amplitude (but if anything a slight facilitation).

The ANOVA for MEP_bi_ amplitude yielded a main effect of *preconditioning* (F_(1,25)_ = 7.71, P = 0.01) but not of *time* (for MEP_bi_), and the ANOVA for MEP_mo_ amplitude only showed a trend (F_(2, 25)_ = 3.46, P = 0.07). Again, there was no impact of *polymorphism* (all P>0.13). A post-hoc two-sided paired t-test for MEP_bi_ revealed that the amount of induced inhibition was larger for cTBS_noTBS_ than for cTBS_cTBS_ (T_26_ = 2.27, P = 0.01).

## Discussion

This study yielded three main findings. First, in the absence of priming iTBS and cTBS consistently induced LTP-like and LTD-like plasticity in M1_HAND,_ as revealed by lasting increases and decreases in mean MEP amplitude. Second, a priming TBS protocol given approx. 30 minutes before consistently primed the plasticity-inducing effects of TBS in a homeostatic fashion. Third, neither the unprimed LTP-like or LTD-like effects of TBS nor homeostatic metaplasticity (as revealed by sequential TBS) were modulated by the individual BDNF val^66^met phenotype. While it is unclear how the absence of a BDNF effect can be explained in comparison to earlier TMS studies (see discussion below), our findings do not support the notion that the val^66^met BDNF polymorphism plays has a major impact on TBS-induced cortical plasticity in the human M1_HAND_.

### LTP-like and LTD-like Plasticity Induced by Unprimed TBS

Replicating previous studies [5], [40], [41], the unprimed TBS interventions reliably induced bi-directional changes in corticospinal excitability of M1_HAND_ as indexed by mean MEP amplitude. While iTBS induced LTP-like increases in mean MEP amplitude, cTBS produced LTD-like decreases. With respect to temporal pattern, stimulus configuration, and stimulus intensity, we used the original TBS protocol as published by Huang et al. (2005) [5]. However, in contrast to Huang et al., we inverted the direction of the induced tissue current, with the second (reversal) phase of the biphasic pulse, which is physiologically most effective, inducing a p-a current in M1_HAND_. Flipping the current direction is relevant from a neurophysiological perspective, because the current direction has a strong impact on the descending volleys that are elicited in the corticospinal tract by a single TMS pulse [42], [43]. A p-a current preferentially recruits early I-waves, whereas an a-p current more readily evokes late I-waves [43], [44], [45], [46]. Since early and late I-waves are thought to be generated by different intracortical circuits [42], our TBS protocol preferentially targeted a different set of cortical circuits as previous TBS studies. Further, a reversal of current orientation has an influence on the stimulus intensity used for TBS which is commonly adjusted to the AMT. This is because the AMT is lower when the second phase of the biphasic pulse elicits a p-a current in M1_HAND_ as opposed to an a-p current [47], [48].

Talelli et al. (2007) [49] directly compared the efficiacy of cTBS and iTBS using either the ‘standard’ (i.e., a-p current for second half wave) or ‘reversed’ (i.e., p-a current for second half wave) current flow with AMT determined with the same respective current directions. While reversed cTBS was ineffective at 80% AMT, it turned out to be even more effective than standard cTBS (80% AMT) when applied at 100% AMT (i.e., matching standard 80% AMT with respect to absolute stimulation intensity). Reversed iTBS at 100% AMT, however, was ineffective or at least not more effective than standard iTBS at 80% AMT. In contrast, Zafar et al. (2008) did not observe significant differences between a-p and p-a current direction when comparing either cTBS or iTBS with different waveforms and current directions at 80% AMT (determined with the respective current direction) [50]. The findings of Zafar et al. are supported by our results, as cTBS and iTBS with p-a current for the second half wave of the biphasic pulse produced clear MEP inhibition and facilitation at 80% AMT, respectively.

On the other hand, a recent study by Hamada et al. (2012) used the standard TBS protocol in 52 subjects and found no consistent overall changes in corticospinal excitability after cTBS and iTBS due to substantial inter-individual variability [51]. Interestingly, however, the TBS effect was highly correlated with the MEP latency evoked by TMS pulses inducing an a-p current in M1_HAND,_ and it was suggested that the inter-individual differences in TBS-induced plasticity depend on which interneuron networks are preferentially excited by the TMS pulse [51]. Although we found considerable variation with respect to the magnitude of TBS-induced after effects, the sign of TBS-induced excitability changes (LTP-like effects induced with iTBS and LTD-like effects induced by cTBS) was highly consistent across subjects with our modified TBS protocol. Our data demonstrate that TBS using a biphasic pulse configuration with its second (most effective) phase inducing a p-a current in M1_HAND_ can consistently induce bidirectional effects on motor cortical plasticity that are at least not inferior to the classical TBS protocol. Moreover, TBS-induced MEP amplitude changes as assessed by monophasic (10 min post-TBS) and biphasic (5 and 25 min post-TBS) pulses were highly comparable, suggesting that both pulse forms are suitable to capture the after effects of TBS on corticospinal excitability (as long as consistently used for pre- and post-TBS measurements).

### Metaplastic Interactions between Two Subsequent TBS Protocols

During the last decade, a growing body of evidence has shown that cortical plasticity as induced by various transcranial stimulation protocols is controlled by homeostatic mechanisms [3]. This homeostatic regulation of LTP-like or LTD-like plasticity (referred to as homeostatic metaplasticity) follows the predictions of the Bienstock–Copper-Munro (BCM) theory [52]. The theory postulates a sliding threshold for LTP/LTD induction, which changes as a function of the history of postsynaptic neuronal activity. It predicts that the threshold for LTP induction increases if the level of activity was high in the past but decreases if it was low. Therefore, a protocol that usually induces LTP may no longer do so or even induce LTD if the level of previous activity has exceeded a certain level. Conversely, a protocol that usually induces LTD may no longer do so or even induce LTP if the level of previous activity has been lowered to a certain level.

These homeostatic mechanisms can be studied in the human M1_HAND_ by priming one plasticity-inducing transcranial stimulation protocol with another one. The majority of studies has explored the interaction between two experimental manipulations targeting different cortical circuits. For instance, the individual response to continuous rTMS was modulated in a homeostatic fashion by a priming session of continuous rTMS [15] or tDCS [13], [14]. Homeostatic metaplasticity was also successfully induced by a priming intervention applied over a remote but inter-connected cortical area such as ipsilateral premotor cortex [53] or contralateral M1_HAND_ [54].

If the homeostatic interaction is examined by using two transcranial stimulation protocols that target different cortical circuits, it remains unclear whether homeostatic interactions occurred within the same (i.e., homosynaptic metaplasticity) or between different cortical circuits (i.e., heterosynaptic metaplasticity [55]. Several recent studies have therefore employed the same interventional paradigm for priming and probing the homeostatic regulation of stimulation-induced LTP-like/LTD-like plasticity in M1_HAND_, including tDCS-tDCS, PAS-PAS, or TBS-TBS [56], [57], [58], [59], [60], [61], [62]. Since these interventional conditioning-test protocols target the same intracortical circuits, they are likely to reflect homeosynaptic metaplasticity. In the present study, we decided for a sequential TBS-TBS intervention to probe homeosynaptic metaplasticity to stimulate the same set of cortical neurons during priming and testing. Furthermore, TBS uses a very low stimulus intensity which ensured a rather focal and selective stimulation of the M1_HAND_.

In good agreement with the BCM theory [52], the effects of TBS on M1_HAND_ excitability critically depended on the priming TBS protocol: If iTBS was primed by cTBS, it produced a stronger LTP-like increase in MEP amplitude than iTBS alone. In contrast, the LTP-like effect on corticospinal excitability was almost completely suppressed if iTBS was primed by iTBS. Likewise, when cTBS was conditioned by cTBS, the primed cTBS failed to induce any additional decrease in mean MEP amplitude. Complementing our results, Todd et al. (2009) showed that the LTD-like effect of cTBS can be enhanced by priming cTBS with iTBS [60]. A homeostatic response pattern to TBS was also demonstrated in a recent study which measured the gain in MEP amplitude with increased stimulus intensity (i.e., the stimulus-response curve) [62]. Relative to the after effects of non-primed TBS, pairing of identical protocols (iTBS-iTBS or cTBS-cTBS) resulted in suppression of the non-primed TBS effects on the stimulus-response curve, whereas pairing of different protocols (cTBS-iTBS or iTBS-cTBS) enhanced the effects of the second TBS on the MEP stimulus-response curve relative to non-primed TBS. Interestingly, TBS also had priming effects on the stimulus-response curve of short-latency intracortical inhibition which correlated with the priming effects on MEP amplitude. These findings suggest that priming effects on intracortical inhibitory circuits might contribute to the homeostatic regulation of metaplasticity of corticospinal motor output [62], [63].

Another recent study systematically varied the interval between two identical TBS protocols. Gamboa et al. (2011) applied either c-cTBS or i-iTBS to M1_HAND_ separated by an interval of 2, 5 or 20 minutes [59]. A homeostatic suppression of the plasticity-inducing after effects of TBS was observed for i-iTBS with an inter-TBS interval of 5 and 20 minutes and for c-cTBS with an inter-TBS interval of 2 and 5 minutes. Together, these results show that two consecutive TBS protocols can be used to study the expression of homeostatic metaplasticity within the same motor cortical circuits in human M1_HAND_. However, the selection of an appropriate interval between the priming and test TBS might be critical. Notably, in our study an even longer inter-TBS interval of approximately 30 min was still effective in producing metaplastic effects. In accordance with the notion of homeostatic metaplasticity, recent results even suggest that the muscle contraction usually preceding TBS interventions during individual AMT determination may have an impact on subsequent TBS efficacy [64]. Also the homeostatic TBS-TBS effects in MEP amplitude as assessed by monophasic (10 min post-TBS) and biphasic (5 and 25 min post-TBS) were highly comparable, suggesting that both pulse forms can be used to study homeostatic TBS-TBS effects.

### No Effect of the val^66^met BDNF Genotype on TBS-induced Plasticity and Metaplasticity

We were not able to replicate a significant effect of the BDNF val^66^met polymorphism on the plasticity-inducing effects of TBS. In contrast to the study by Cheeran et al. (2008) [29], val^66^met carriers showed a consistent LTP-like effect after a single iTBS session as well as a consistent LTD-like effect after a single cTBS session, and the magnitude of the observed changes in MEP amplitude were comparable to the effects observed in val^66^val allele carriers. Furthermore, the BDNF val^66^met polymorphism had no influence on the homeostatic response pattern evoked by a priming TBS intervention. This finding is also at variance with the study by Cheeran et al. (2008) [29] in which val^66^met allele carriers lacked the normal homeostatic effect of cathodal tDCS on subsequent 1 Hz rTMS. While only Cheeran et al. (2008) [29] and the present study have investigated the impact of the val^66^met BDNF genotype on the individual response to cTBS as well as on homeostatic metaplasticity, two additional studies have in fact examined its influence on the effects of iTBS. Again, the results were conflicting. Antal et al. (2010) reported that LTP-like plasticity could only be induced in 10 val^66^val allele carriers but not in 5 val^66^met allele carriers with iTBS [37], but Li Voti et al. (2011) found no difference between 7 val^66^met and 14 val^66^val allele carriers in their response to iTBS [36].

How can these inconsistencies be explained? While subjects’ age (between 20 and 30 years in all studies) or TBS stimulation intensity (always 80% AMT) were comparable between studies, several other experimental parameters differed and may have contributed to the divergent results. First, regarding the effect of BDNF polymorphism on homeostatic metaplasticity it should be noted that we used a different protocol than Cheeran et al. [29] which might be less sensitive to the val66met BDNF genotype. The TBS-TBS and tDCS-1Hz rTMS protocol test homeosynaptic and heterosynaptic metaplasticity, respectively, and thus, involve different intracortical circuits.

Second, the direction of current flow during iTBS may be relevant as it results in a preferential stimulation of different cortical circuits which may be more or less sensitive to variations in the BDNF val66met genotype. However, since the direction of current flow in the brain tissue induced by the second (most effective) phase of the biphasic TMS pulse was a-p in Cheeran et al. [29] and Li Voti et al. [36], whereas it was p-a in Antal et al. [37] and the present study, it might not be the crucial parameter determining whether a BDNF effect can be observed.

Third, whether or not homocygous met^66^met carriers were included in addition to heterozygous val^66^met carriers may be relevant as a previous study suggested a “dose” effect of the BDNF val^66^met polymorphism on memory function [65]. Indeed, only Cheeran et al. [29] and Antal et al. [37] included one and three met66met carriers, respectively. However, it is unlikely that the observed BDNF effects in these studies were driven by these few extreme subjects.

Fourth, the gender distribution of the study sample may be relevant. This is not trivial considering that ovarian hormones can influence cortical excitability and Inghilleri et al. (2004) reported that estrogen levels lowered cortical excitability by acting on sodium channels, dampening down the recruitment of excitatory interneurons, and thereby reducing cortico-spinal facilitation [24]. Our sample consisted of men only, while the three other studies included a comparable number of male and female participants. Unfortunately, none of them tested for gender differences.

Fifth, the TMS intensity during MEP measures may be important as different intracortical circuits are probed at low and high intensities. However, baseline MEP amplitudes (around 0.61 mV in our study, around 0.81–0.97 mV in Cheeran et al. [29], and around 1.0 mV in Li Voti et al. [36] and Antal et al. [37] do not seem to predict whether or not a BDNF effect was found.

Sixth, the impact of the BDNF polymorphism might be expressed differentially across various post-TBS intervals. While we acquired MEP measures starting at 5, 10, and 25 min post-TBS, Cheeran et al. [29] did so at 1–5, 6–8, 9–11, 12–15 and 16–24 min, Li Voti et al. [36] at 5, 15, and 30 min, and Antal et al. [37] at 0, 5, 10, 15, 20, 25, 30, and 60 min after the end of TBS. However, neither Cheeran et al. [29] nor Antal et al. [37], report any significant interaction between the BDNF polymorphism and the timing of post-TBS measurement. Further, visual inspection suggests effects being visible throughout the first 25 min post-TBS, the time period which was covered by Li Voti et al. [36] and our measurements as well.

Seventh, the TMS pulse form used for MEP measurements might be relevant because different intracortical circuits are targeted by monophasic and biphasic pulses. While we used both biphasic (5 and 25 min) and monophasic (10 min) pulses, all other studies only used monophasic pulses to assess TBS effects on MEP amplitude (the relevant current flow in the brain was always in p-direction for all studies). However, neither we nor Li Voti et al. [36] were able to detect an effect of BDNF polymorphism on TBS-induced after effects, when using monophasic pulses. In summary, none of the discussed study parameters can easily account for the observed divergence of results regarding TBS-induced changes in corticospinal excitability.

The effect of BDNF val^66^met polymorphism has also been tested using other plasticity-inducing protocols: Two studies found that the BDNF val^66^met polymorphism influences the LTP-inducing effects of PAS: In 9 val^66^val allele carriers, PAS at an interval of 25 ms (PAS-25) produced a significant increase in MEP amplitude, whereas PAS-25 had no significant effect in 9 val^66^met allele carriers. [29], [37]. The same pattern was reported in a small sample of 14 twins, consisting of 10 val^66^val, 2 val^66^met, and 2 met^66^met allele carriers [28]. Another study found no difference in the response to PAS-25, when the BDNF val^66^met genotype was considered in isolation, but there was a interaction between the BDNF val^66^met genotype and the catechol-O-methyltransferase (COMT) val1^58^met genotype [39]: In individuals with a val^66^val BDNF genotype, PAS-25 ms only had a stronger LTP-like effect when subjects also had a met^58^met COMT genotype. No influence of the BDNF val^66^met polymorphism has been reported for 5Hz rTMS [36], quadruple-pulse stimulation [35], or transcranial random noise stimulation [37]. With respect to tDCS, anodal and cathodal tDCS have been reported to be more effective in individuals carrying a met-allele in the BDNF gene relative to homocygous val^66^val allele carriers [37] which is opposite to the genotype effect reported for PAS and TBS. However, Cheeran et al. (2008) [29] found a similar response of val^66^met and val^66^val allele carriers to cathodal tDCS.

### Conclusion

The data suggest that the BDNF val^66^met polymorphism may have an impact on the plasticity-inducing effects of transcranial stimulation protocols, but the effects seem to be variable and to critically depend on the specific plasticity-inducing protocol. Although some studies have reported dramatic differences in the individual response to stimulation protocols such as TBS or PAS, it is questionable whether the individual BDNF val^66^met genotype is a useful genetic marker for predicting plasticity-inducing effects of transcranial brain stimulation in a given subject. To derive a robust estimate of the real impact of the BDNF val^66^met genotype on stimulation-induced plasticity, the effects need to be assessed in multi-center studies on larger cohorts. It is, however, unlikely that such studies will identify the BDNF val^66^met genotype as a key factor. Many other genetic variations influencing ion channel function or neurotransmitter release may modify the plasticity-inducing effects of non-invasive brain stimulation and the impact of the individual BDNF val^66^met genotype [39]. For instance, a functional single nucleotide polymorphism in the gene coding the transient receptor potential vanilloid 1 (TRPV1) channels which regulate glutamate release was shown to account for inter-individual differences in short-interval intracortical facilitation [66]. Although this particular variation in the TRPV1 gene did not influence the individual plasticity response to cTBS or iTBS, it is conceivable that genes controlling synaptic transmission, and thereby, the dynamics of post-synaptic Ca^2+^ influx have an influence on the plasticity-inducing effects of transcranial brain stimulation protocols in intracortical networks. Indeed, a recent pharmacological TBS study provided strong support that the Ca^2+^ dynamics determine the direction of LTP/LTD-like plasticity as induced by TBS in human M1 [67].

In addition to manifold genetic factors that regulate the induction of synaptic plasticity, the “neural state” at the time of transcranial conditioning is highly relevant. For instance, changes in attention at the time of transcranial stimulation exert a powerful modulatory effect on the plasticity-inducing effects of rTMS protocols, including TBS and PAS [68], [69]. Furthermore, a recent study has provided evidence that the inter-individual variation in the neuroplastic response to rTMS protocols might depend on which intracortical networks are preferentially recruited by the TMS pulse [51]. If so, inter-individual variations in genes that regulate the expression of plasticity in cortical synapses constitute only one of many factors that shape the individual response to interventional transcranial stimulation. Given the multitude of genetic and non-genetic factors, a normal functional variation in a single gene might only play a relatively minor role in determining the neuroplastic effects of transcranial brain stimulation protocols.
